# Molecular Characterization of Arabinoxylans from Hull-Less Barley Milling Fractions

**DOI:** 10.3390/molecules16042743

**Published:** 2011-03-24

**Authors:** Xueling Zheng, Limin Li, Xiaoxi Wang

**Affiliations:** Grain College, Henan University of Technology, Zhengzhou 450052, Henan Province, China; E-Mails: lilimin1970@126.com (L.L.); xiaoxiwang@haut.edu.cn (X.W.)

**Keywords:** arabinoxylan, hull-less barley, size exclusion chromatography analysis, dynamic light scattering analysis

## Abstract

Arabinoxylans were prepared from different hull-less barley milling fractions (bran, shorts and flour). The yields of hull-less bran arabinoxylan (HBB-AX), shorts arabinoxylan (HBS-AX) and flour arabinoxylan (HBF-AX) were 8.42%, 4.08% and 2.13% respectively. Sugar composition analysis showed that arabinose and xylose were the main sugars. HBF-AX had the highest Ara/Xyl ratio, followed by HBS-AX and HBB-AX. Size exclusion chromatography analysis (HPSEC) showed that HBF-AX had the highest molecular weight, followed by HBS-AX and HBB-AX, which had the lowest molecular weight. Aqueous solutions of HBB-AX, HBS-AX and HBF-AX had higher molecular weight(Mw) than in 0.5 mol/L NaOH and 1.0 mol/L NaOH. Dynamic light scattering analysis (DLS) showed that HBB-AX, HBS-AX and HBF-AX had existed in two states in distilled water, 0.5 mol/LNaOH, 1.0 mol/L NaOH, and DMSO/H_2_O (90/10), an unaggregated state and an aggregated state, with the latter predominating.

## 1. Introduction

Hull-less barley has recently attracted a lot of interest among food scientists and technologists because of its soluble dietary fiber, β-glucan and arabinoxylan (AX) content in particular. The intake of β-glucan and arabinoxylan from hull-less barley has considerable health benefits [1,2]. The amount of arabinoxylan in hull-less barley normally varies between 3% to 7% [3]. This level of arabinoxylan is influenced by both genetic and environmental factors, but genetic factors appear to be of greater importance [4]. The current interest in barley and oat soluble dietary fiber stems from evidence for reduction of serum cholesterol and glucose levels, as well as reduction of the associated risks for chronic diseases, when consumed in the human diet [5,6]. Arabinoxylans are found in the cell walls of barley, oat, wheat, rye, maize rice, sorghum, and millet [7]. Arabinoxylan is constituted of a linear backbone of β-(1→4)-linked D-xylopyranosyl units to which α-L-arabinofuranosyl substituents are attached through O-2 and/or O-3. Some of the arabinose residues are ester linked on (O)-5 to ferulic acid (FA) [8].

Arabinoxylan can be prepared by hot water and alkaline solutions, such as NaOH, KOH, saturated Ba(OH)_2_ and Ca(OH)_2_ [9,10]. Arabinoxylans prepared with hot water have higher Ara/Xyl ratios and lower molecular weights compared with arabinoxylans prepared in alkaline solutions [11]. Molecular characterization of arabinoxylans by high-performance size-exclusion chromatography (HPSEC) has been broadly researched [12]. For analysis of the molecular characteristics, complete dissolution of arabinoxylan in a suitable medium is important. Distilled water, dimethyl sulfoxide (DMSO) and aqueous solutions of inorganic alkali are commonly used to dissolve polysaccharides [13,14]. Macromolecular aggregates are inevitably present due to the numerous intermolecular hydrogen bonds formed between polysaccharide molecules in dilute solution [12,15]. Dynamic light scattering (DLS) is an effective approach to study the aggregation behavior of polysaccharide in dilute solutions, and can distinguish the aggregated from the unaggregated state [16].

Hull-less barley can be dry-milled and separated into flour and bran fractions using conventional roller-milling technology [17]. Although hull-less barley has not traditionally been roller-milled like wheat to obtain flour and bran, this may change in the near future because of hull-less barley's high soluble fiber content and its potential use in many food products. Apart from β-glucan, arabinoxylan is another major component of the dietary fiber in hull-less barley [1], its level and distribution in hull-less barley roller-milled fractions will give an indication of the potential nutritional qualities of the milled products. Therefore, the objectives of this study are to prepare arabinoxylans from hull-less barley roller-milled fractions and study their molecular properties, and the formulation of hull-less barley milling fractions targeted for specific applications.

## 2. Results and Discussion

### 2.1. Chemical composition of hull-less barley bran, shorts and flour

Hull-less barley was milled in a Buhler roller-mill to obtain bran, shorts and flour fractions. The yields were 18.1%, 10.6% and 71.3% respectively, and their chemical composition is shown in Table 1. The bran and shorts fractions contained higher concentrations of ash, protein, β-glucan, arabinoxylan, and lower concentration of starch than the flour fraction. The distribution of arabinoxylan in the three roller-milled fractions of hull-less barley was uneven. Bran contained the highest concentration of (10.26%), followed by shorts (4.65%) and flour (3.17%), which indicated that in the hull-less barley kernels the arabinoxylan was mainly distributed in the outer bran part (the main component of bran), followed by the aleurone part (the main component of shorts). The endosperm part (the main component of flour) had the lowest arabinoxylan concentration, which has also been reported previously [3,18]. 

**Table 1:** Composition of hull-less barley (HB) bran, shorts and flour.

Component	HB bran (%,db)	HB shorts (%,db)	HB flour (%,db)
Ash	3.83	2.23	0.74
Crude protein	17.42	14.76	11.80
Starch	25.62	45.72	69.02
Arabinoxylan	10.26	4.65	3.17
β-Glucan	7.75	10.76	5.03
Neutral monosaccharides			
Ara	4.42	2.15	1.52
Gal	1.32	1.50	0.56
Glu	33.66	57.08	72.43
Xyl	7.24	3.13	2.08
Ara/Xyl	0.61	0.69	0.73

Results are expressed as averages of duplicates. The deviation in composition was within a 5% limit. Ara: arabinose; Gal: galactose; Glu: non-cellulosic glucose. Sugars expressed as weight percentage of each fraction. Total arabinoxylan= 0.88×(%Ara +%Xyl).

### 2.2. Composition of arabinoxylans from hull-less barley bran, shorts and flour

Different alkaline solutions have been reported for the preparation of arabinoxylans from cereals, such as different concentrations of NaOH (0.5, 1.0 or 2.0 mol/L), saturated Ba(OH)_2_ and Ca(OH)_2_. Arabinoxylans that were prepared with saturated Ba(OH)_2_ had higher purity and lower recovery than those prepared using NaOH solutions [19,20].

**Table 2:** Chemical composition of HBB-AX, HBS-AX, and HBF-AX.

		HBB-AX	HBS-AX	HBF-AX
Yield ^a^ (%)		8.42	4.08	2.13
Composition^ b^ (%, db)				
Ash		2.06	2.34	2.06
Crude protein		4.26	5.64	5.38
Neutral monosaccharides after hydrolysis				
	Ara	31.80	34.71	36.01
	Gal	1.81	1.19	1.37
	Glu	2.11	3.21	1.04
	Xyl	58.19	52.80	47.28
Arabinoxylan ^c^		79.19	77.01	73.30
Ara/Xyl		0.55	0.66	0.76

a Expressed as weight percentage(dm) of hull-less barley flour, bran, and shorts. b Based on 100g of each fraction (dm). c Total arabinoxylan= 0.88×(%Ara +%Xyl).

Arabinoxylan recovery could be increased by increasing the NaOH solution concentration, but higher NaOH concentrations could degrade arabinoxylan at room temperature, and it was concluded that the optimum conditions for preparation minimally degraded arabinoxylan from cereal bran was 0.5 mol/L NaOH at room temperature [20]. In this research, arabinoxylans were prepared from hull-less barley bran (HBB), shorts (HBS), and flour (HBF) using 0.5 mol/L NaOH at room temperature. The yield of HBB-AX (8.42%) was higher than that of HBS-AX (4.08%) and HBF-AX (2.13%) (Table 2). Detailed analysis of the neutral sugar composition of HBB-AX, HBS-AX and HBF-AX showed that arabinose and xylose were the main sugars. HBB-AX, HBS-AX, and HBF-AX had different Ara/Xyl ratios. HBF-AX had a higher Ara/Xyl ratio (0.76) compared with HBB-AX (0.55) and HBS-AX(0.66), which was not agreement with previous a report that the Ara/Xyl ratio of arabinoxylan prepared from cereal endosperm was usually lower (0.50-0.71) than that from cereal bran (1.02-1.07) [18]. Perhaps the difference in our case was due to the properties of the raw material and preparation methods.

### 2.3. Molecular characterization of HBB-AX, HBS-AX and HBF-AX by HPSEC

HBB-AX, HBS-AX and HBF-AX were dissolved in distilled water, 0.5 mol/L NaOH and 1.0 mol/L NaOH, respectively, in order to compare the effects on the molecular properties of the arabinoxylans. The results are shown in Table 3. The range of molecular weight of the arabinoxylans in the studied hull-less barley was 276,000 to 877,100 g/mol, which was in agreement with previous reports [21,22].

**Table 3:** Molecular weight(Mw), radius gyration (Rg), intrinsic viscosity [η], and polydispersity (Pd) of HBB-AX, HBS-AX and HBF-AX in different solvents.

	Mw (g/mol)	Rg (nm)	[η] (dL/g)	Pd	Recovery (%)
HBB-AX					
Water solution	369,000	37.01	4.16	1.82	76
0.5 mol/L NaOH	318,200	32.22	3.29	1.20	83
1.0 mol/L NaOH	276,000	30.60	3.29	1.19	92
HBS-AX					
Water solution	484,000	38.50	4.28	2.06	81
0.5 mol/L NaOH	457,300	38.09	4.06	1.68	89
1.0 mol/L NaOH	405,000	32.06	3.19	1.92	95
HBF-AX					
Water solution	877,100	58.60	7.17	1.27	67
0.5 mol/L NaOH	758,200	54.73	6.13	1.04	79
1.0 mol/L NaOH	604,000	48.27	5.50	1.19	89

Mw, weight average molecular weight; Rg, radius of gyration; [η], intrinsic viscosity; Pd, the ratio of weight-average molecular weight to number average molecular weight (M_w_/M_n_)

Arabinoxylans from flour (HBF-AX) yielded the highest Mw compared to those from shorts (HBS-AX) and bran (HBB-AX), and arabinoxylans from bran had the lowest Mw, which was similar to that of β-glucans from hull-less barley flour, bran and shorts [3]. The molecular weight difference of HBB-AX, HBS-AX and HBF-AX indicated that the arabinoxylans in different part of the hull-less barley kernels had different structural properties. HBB-AX, HBS-AX and HBF-AX had higher molecular weight in water solution than in 0.5 mol/L NaOH and 1.0 mol/L NaOH. Arabinoxylans had the lowest molecular weight in 1.0 mol/L NaOH solution, suggesting that 1.0 mol/L NaOH could degrade the arabinoxylans, as reported previously [20]. The ratio of weight-average molecular weight to number-average weight ((M_w_/M_n_ or Pd) of HBB-AX, HBS-AX and HBF-AX ranged from 1.04 to 2.06, indicating the three arabinoxylans had a broad molecular weight distribution, which is in good agreement with a previous report [18], that found Pd values ranging from 1.3 to 2.5 for alkali-extractable arabinoxylans. The radius of gyration (Rg) and intrinsic viscosity ([η]) of HBF-AX were higher than those of HBB-AX and HBS-AX. From the recovery of HBB-AX, HBS-AX and HBF-AX (Table 3), it could be seen that 1.0 N NaOH had good solvating effect for arabinoxylans. Water heating treatment (70°C for 2 h, then stirring overnight at room temperature) had worse solvating properties for arabinoxylans compared with 0.5 N NaOH and 1.0 N NaOH solutions (25°C, 2 h). 

### 2.4. Aggregation properties of HBB-AX, HBS-AX and HBF-AX in dilute solution measured by DLS

**Figure 1:**
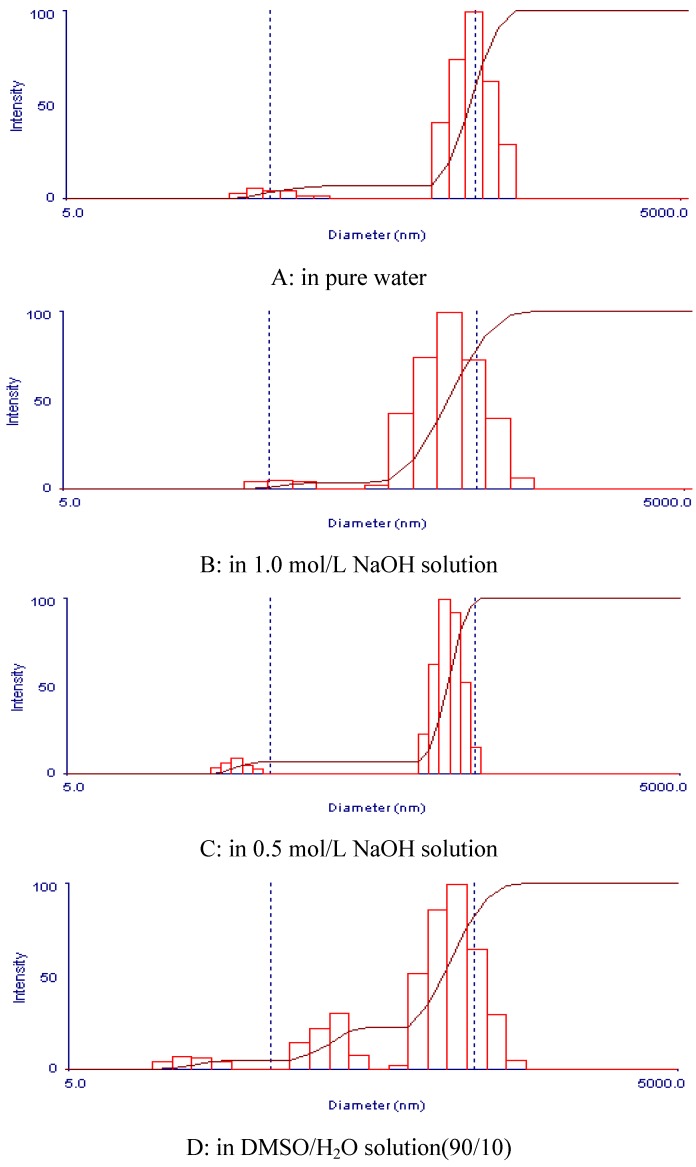
The molecular size distribution of HBB-AX by DLS in pure water, 0.5 mol/L NaOH, 1.0 mol/L NaOH and DMSO/H_2_O (90/10).

The particle size distribution of HBB-AX, HBS-AX and HBF-AX in distilled water, 0.5 mol/L NaOH, 1.0 mol/L NaOH and DMSO/H_2_O (90/10) are shown in Table 4 and Figure 1. Two states, the unaggregated state and the aggregated state existed, and HBB-AX, HBS-AX and HBF-AX in water solution, 0.5 mol/L NaOH, 1.0 mol/L NaOH, or DMSO/H_2_O (90/10), mainly existed in an aggregated state, in agreement with previous reports [23,24].

**Table 4:** Dynamic light scattering results of HBB-AX, HBS-AX and HBF-AX in different solutions.

	Mean diameter (nm)		
	Total average	Non-aggregates	Aggregates
**HBB-AX**			
Water solution	456.6	53.5	484.1
0.5 mol/L NaOH	333.4	49.2	362.4
1.0 mol/L NaOH	183.3	29.8	226.7
DMSO/H_2_O	341.6	79.5	366.5
**HBS-AX**			
Water solution	331.3	67.0	341.5
0.5 mol/L NaOH	354.9	36.4	371.5
1.0 mol/L NaOH	285.6	43.5	312.9
DMSO/H2O	221.3	42.3	238.2
**HBF-AX**			
Water solution	341.6	46.6	351.9
0.5 mol/L NaOH	373.5	57.0	387.4
1.0 mol/L NaOH	383.2	39.4	409.5
DMSO/H2O	248.5	60.6	272.3

The results indicated that macromolecular aggregates were inevitably present due to the numerous intermolecular hydrogen bonds among arabinoxylan molecules. Many researchers have reported the presence of macromolecular aggregates in dilute polysaccharide solutions [15,25]. The results showed that some other physical or chemical methods to eliminate the aggregation of arabinoxylans should be studied. To this end some research has found that heat treatment, filtration, ultrasonication, and use of urea solutions could reduce the aggregate of polysaccharides [23,24].

HBB-AX had a larger molecular size distribution in water solution, the average molecular size was 456.6 nm, and HBB-AX was mainly existed in an aggregated state. The molecular size for HBB-AX in non-aggregated state was 53.5 nm. The average molecular size was smaller in 0.5 mol/L NaOH solution (333.4 nm) and 1.0 mol/L NaOH solution (183.28 nm), which indicated that alkali could reduce the aggregation of HBB-AX by breaking the intermolecular hydrogen bonds among arabinoxylan molecules. Although DMSO/H_2_O (90/10) has been reported to a good solvent to arabinoxylan and other macromolecules [24], it could not eliminate the aggregate of arabinoxylan.

## 3. Experimental

### 3.1. Materials and Chemicals

Hull-less barley was obtained from Lanzhou Qizheng Group (Gansu Province, China) during the 2008 crop year. The barley cultivar was Zangqing, a widely cultivated hull-less barley variety in Tibet. Heat stable α-amylase, lichenase were purchased from Megazyme International (Bray, Co. Wicklow, Ireland). All chemicals were of reagent grade. 

### 3.2. Hull-less barley milling

The moisture of the hull-less barley was adjusted to 16%, and it was then conditioned for 30 hr at room temperature (about 25°C). The conditioned hull-less barley was then milled using a Buhler laboratory mill (Buhler MLU 202, Buhler Corp, Switzerland). The laboratory mill has three break systems and three middling systems. The feeding was adjusted to 15-20 min/kg during milling. Six flour fractions from break and middling systems were obtained. The six fractions from the starchy endosperm were combined to give a flour fraction. Bran and shorts fractions were also collected. 

### 3.3. Preparation arabinoxylans from hull-less bran, shorts and flour

**Figure 2:**
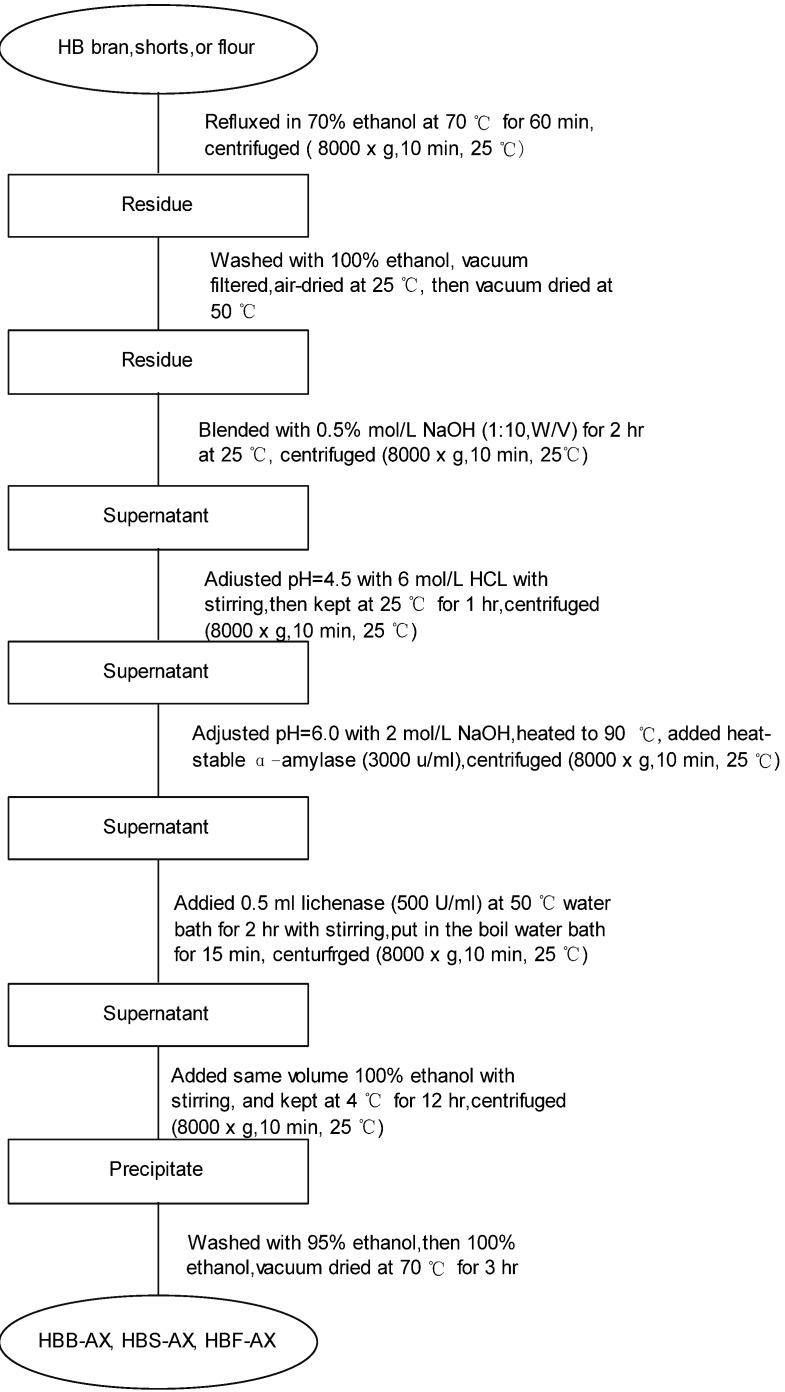
Preparation of arabinoxylans from hull-less bran and shorts and flour

Arabinoxylans were prepared from hull-less bran, shorts and flour according to Cui with some modification [20] (Figure 2). Hull-less bran was ground to pass 40 mesh. Hull-less bran, shorts or flour was refluxed in 70% ethanol. The ethanol refluxed hull-less bran, shorts or flour was extracted with 0.5 mol/L NaOH, and the alkaline supernatant was adjusted to pH=4.5 with 6 mol/L HCl to remove protein. After centrifugation, the supernatant was adjusted pH=6.0 and incubated with heat-stable α-amylase (3,000 units in 2 mL of water, Termamyl 1201, Novo Nordisk, Denmark) to remove soluble starch, and then incubated with lichenase (500U/mL) to remove β-glucan. The supernatant was precipitated with ethanol to obtain the hull-less bran, shorts and flour arabinoxylans (HBB-AX, HBS-AX and HBF-AX). 

### 3.4. Analytical methods

#### 3.4.1. Chemical analyses

Moisture and ash contents were measured by AACC methods 44-15A and 08-01 respectively. Crude protein was analyzed by NA2100 Nitrogen & Protein Analyzer using the factor of 5.7 for hull-less barley flour and 6.25 for bran and shorts to convert nitrogen to protein. The β-glucan content was determined by the specific enzyme method [26] using reagents supplied by Megazyme International (Bray, C0. Wicklow, Ireland).

#### 3.4.2. Monosaccharide analysis

Monosaccharide composition of the arabinoxylans was determined by high-performance anion-exchange chromatography (HPAEC, Dionex system) after hydrolysis with 1 mol/L H_2_SO_4_ for 2 h in a 100°C glycerin bath as described by Wood [27]. The total non-cellulosic sugar content was calculated as the sum of all detected monosaccharides with a conversion factor when pentoses (0.88) or hexoses (0.9) were incorporated in the polymers. The arabinoxylan content was calculated as 0.88×(% arabinose + % xylose) [9].

#### 3.4.3. Molecular characterization by size exclusion chromatography (HPSEC)

In HPSEC analysis of molecular properties of HBB-AX, HBS-AX and HBF-AX, three treatments were studied for their influence on molecular properties: 1) HBB-AX, HBS-AX and HBF-AX were dissolved in pure water in 70 °C for 2 h, stirred overnight at room temperature, then filtered with a 0.45 μm filter, and injected for HPSEC analysis. 2) HBB-AX, HBS-AX and HBF-AX were dissolved in 0.5 mol/L NaOH at room temperature for 2 h with stirring, filtered with a 0.45 um filter, and analyzed by HPSEC. 3) HBB-AX, HBS-AX and HBF-AX were dissolved in 1.0 mol/L NaOH at room temperature for 2 h with stirring, filtered with a 0.45 μm filter, and analyzed by HPSEC.

Molecular weight, radius of gyration and intrinsic viscosity of HBB-AX, HBS-AX and HBF-AX were determined by HPSEC equipped with three detectors: a right angle laser light scattering detector (RALLS), a differential pressure viscometer (DP) and a refractive index detector (RI) (Triple Detector System, Model Dual 250, Viscotek, Houston, TX, USA). The chromatographic system included a Shimadzu SCL-110Avp pump and automatic injector (Shimadzu Scientific Instruments Inc. Columbia, MD, USA), two columns in series: a Shodex Ohpak KB-806M (Showa Denko K.K, Tokyo, Japan), and an Ultrahydrogel linear (Waters Milford, CT, USA). To test the accuracy of the calibration, three pollulan standards (P-100, P-400, and P-800) were used. Recovery of these polysaccharide standards was >95%. The M_w_ values were 110,400 for P-100, 405,800 for P-400, and 767,000 D for P-800. These values were in good agreement with those given by the manufacturer (112,000, 404,000, and 788,000, respectively).

#### 3.4.4. Aggregation properties of HBB-AX, HBS-AX and HBF-AX in dilute solution measured by DLS

The particle size distribution and the aggregating properties of HBB-AX, HBS-AX and HBF-AX in dilute solution were measured by DLS. The DLS measurements were conducted using a Brookhaven light scattering instrument including a precision goniometer, a photomultiplier and a 128-channel BI-9000AT digital autocorrelator (Brookhaven Instruments, Holtsville, NY, USA). The measurements were carried out at a 90 degree angle. The particle size distributions were calculated with the Brookhaven dynamic light scattering software using the non-negatively constrained least squares (NNLS) approach. Four kinds of solvents were used for dissolving arabinoxylans to study their influence on molecular properties. The concentration of arabinoxylans in solution was 0.1 mg/mL. The samples were prepared as follows: 1) HBB-AX, HBS-AX and HBF-AX were dissolved in pure water at 70°C for 2 h, then stirred overnight at room temperature, and filtered with a 0.45 μm filter. 2) HBB-AX, HBS-AX and HBF-AX were dissolved in 0.5 mol/L NaOH solution in room temperature for 2 h with stirring and filtered with a 0.45μm filter. 3) HBB-AX, HBS-AX and HBF-AX were dissolved in 1.0 mol/L NaOH solution in room temperature for 2 h with stirring, and filtered with a 0.45 μm filter. 4) HBB-AX, HBS-AX and HBF-AX were dissolved in DMSO/H_2_O (90/10) solution at room temperature for 2 h with stirring, and filtered with a 0.45 μm filter.

## 4. Conclusions

Hull-less barley were roller-milled to obtain flour (71.3%), shorts (10.6%) and bran (18.1%) fractions. Arabinoxylan, as a major non-starch polysaccharide in hull-less barley kernel, was mainly concentrated in the outer and aleurone parts, which was the major component of hull-less barley bran and shorts. Arabinoxylans from bran, shorts and flour had different Ara/Xyl ratios, with arabinoxylan from bran displaying the highest Ara/Xyl ratio. Arabinoxylans had a very broad molecular weight distribution. Arabinoxylans from hull-less barley bran, shorts, and flour had different molecular properties. Arabinoxylan in hull-less barley endosperm had higher molecular weight, and in the outer part of hull-less barley kernel it had low molecular weight, which would influence their physicochemical properties. Two states, the unaggregated state and the aggregated state existed, and arabinoxylans in dilute solutions mainly existed in the aggregated state. Hull-less barley bran and shorts, as roller-milling by-products, would be reasonable materials to prepare arabinoxylans as health food additives.
